# Supplementary material for: A droplet-based microfluidic platform enables high-throughput combinatorial optimization of cyanobacterial cultivation
Source: Sci Rep. 2022 Sep 15;12:15536. doi: 10.1038/s41598-022-19773-6 (PMC9477827; doi:10.1038/s41598-022-19773-6)
Supplement: Supplementary file 1 — Supplementary Figures. [file 41598_2022_19773_MOESM1_ESM.pdf]

Supplementary Material

A droplet-based microfluidic platform enables high-throughput combinatorial optimization of cyanobacterial cultivation

Jialan Cao[1†], David A. Russo[2], Ting Xie[1], G. Alexander Groß[1], and Julie A. Z. Zedler[3†]

[1]Ilmenau University of Technology, Institute for Chemistry and Biotechnology, Dept. Physical Chemistry and Microreaction Technology, Weimarerstr. 32, D-98693 Ilmenau, Germany
[2]Friedrich Schiller University Jena, Institute for Inorganic and Analytical Chemistry, Bioorganic Analytics, Lessingstr. 8, D-07743 Jena, Germany
[3]Friedrich Schiller University Jena, Matthias Schleiden Institute for Genetics, Bioinformatics and Molecular Botany, Synthetic Biology of Photosynthetic Organisms, Dornburgerstr. 159, D-07743 Jena, Germany

[†]Correspondence: jialan.cao@tu-ilmenau.de; julie.zedler@uni-jena.de

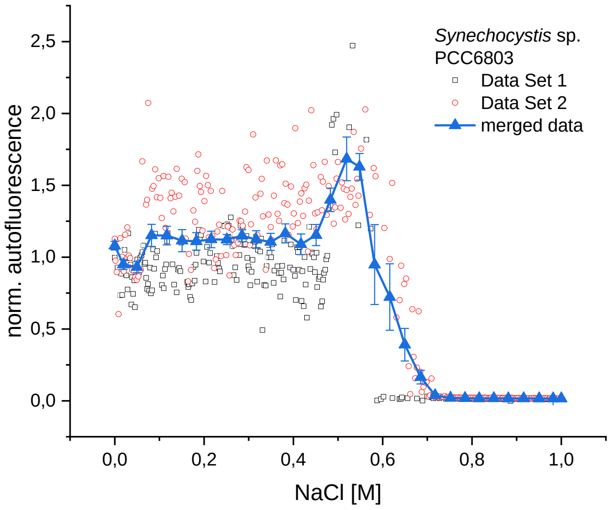

Supplementary Fig. 1 A representative example of how the microdroplet raw data is visualized and treated for each experiment. Details can be found in the Materials &Methods section “Statistics and Reproducibility”.

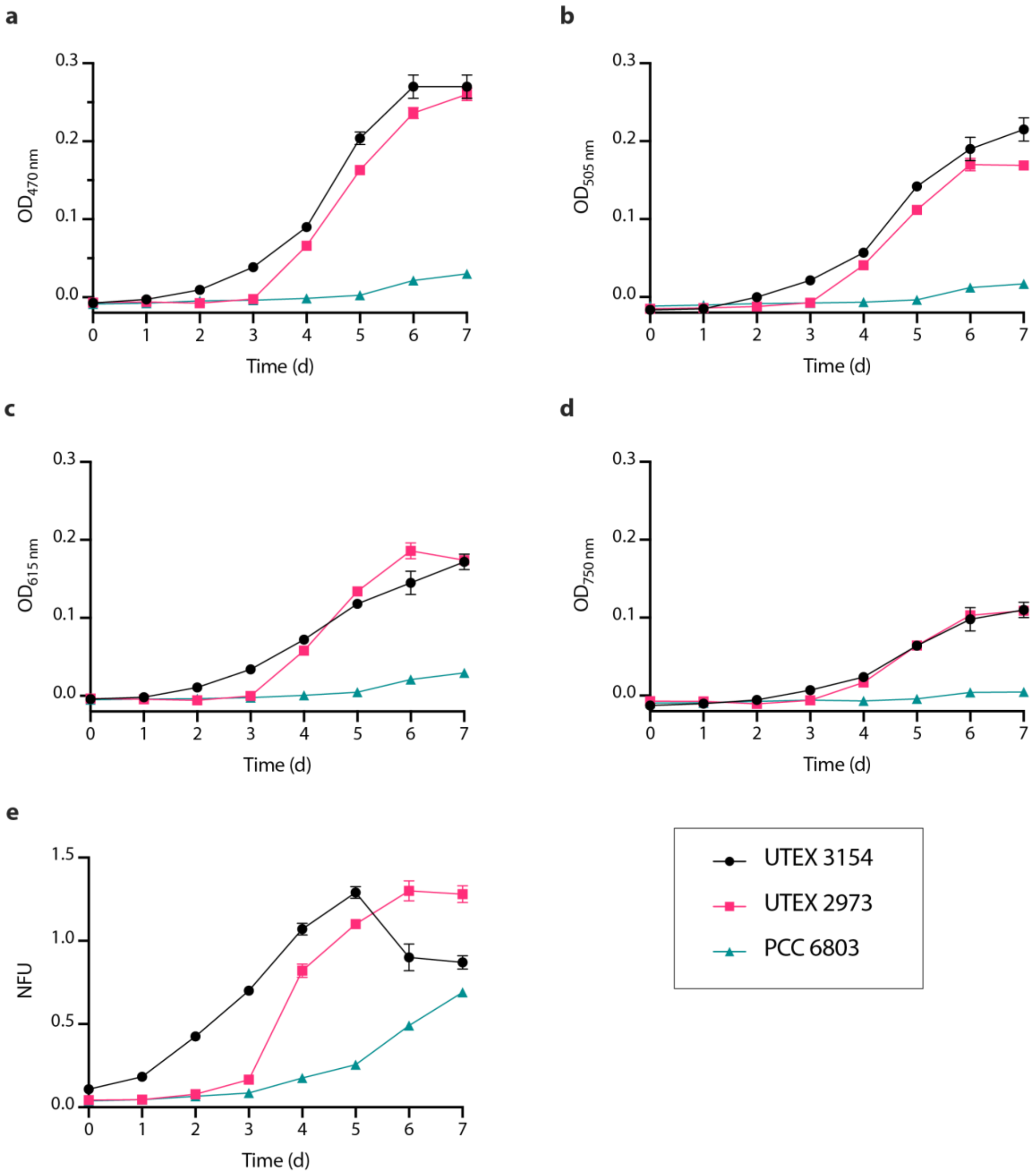

Supplementary Fig. 2 Growth kinetics of three cyanobacterial strains in microdroplet setup over a period of 7 days. Measurements of multi-channel photofluorimeter with different exctinction wavelengths and fluorescence. **a** optical density (OD) at 470 nm, **b** OD at 505 nm, **c** OD at 615 nm, **d** OD at 750 nm and **e** normalized fluorescence units (NFU) (excitation: 405 nm, emission: 425 nm). Data points are averages of approximately 50 droplets. Error bars represent standard deviation.
